# Gonadal hormones and impulsivity facets: moderating roles of cortisol and anxiety

**DOI:** 10.3389/fpsyg.2026.1871253

**Published:** 2026-09-03

**Authors:** Efrat Barel, Ami Cohen, Lila Mahagna

**Affiliations:** 1 School of Psychological Sciences, University of Haifa, Haifa, Israel; 2 Department of Psychology, The Max Stern Academic College of Emek Yezreel, Jezreel Valley, Israel; 3 Laboratory Medicine Department, Emek Medical Center, Afula, Israel

**Keywords:** anxiety, cortisol, estradiol, impulsivity, testosterone

## Abstract

Impulsivity is a multidimensional construct that reflects both affective reactivity and cognitive control processes. Although gonadal hormones have been implicated in impulsive behavior, their associations with specific impulsivity facets may depend on contextual physiological and affective conditions. In this study, we ask whether internal neuroendocrine and psychological states moderate associations between gonadal hormones and impulsivity facets. Salivary levels of testosterone, estradiol, and cortisol are assessed in a mixed sample of 197 participants comprised of 66 men, 68 users of oral contraceptives, and 63 naturally cycling women in the mid-luteal phase. Participants complete the four facets of the Urgency-Premeditation-Perseverance-Sensation (UPPS) Impulsive Behavior scale and a measure of state anxiety. Moderated regression analyses examine whether cortisol levels and anxiety moderate associations between gonadal hormones and impulsivity facets. Results indicate that hormonal associations with impulsivity are context-dependent. Cortisol and anxiety moderate associations between gonadal hormones and regulatory facets of impulsivity. In particular, estradiol shows context-dependent associations with lack of premeditation and lack of perseverance, whereas testosterone is associated with perseverance at higher anxiety levels. These effects are specific to regulatory facets of impulsivity and are not observed for urgency or sensation-seeking. The findings suggest that associations between gonadal hormones and impulsive behavior may be contingent upon concurrent neuroendocrine and psychological contexts.

## 1 Introduction

Impulsivity, defined as a tendency toward rapid and unplanned reactions to specific internal or external stimuli without consideration of long-term consequences (Moeller et al., 2001), has attracted increasing attention in psychology, cognitive neuroscience, and psychiatry because it is increasingly recognized as a transdiagnostic construct that contributes to a wide range of maladaptive behaviors and neuropsychiatric disorders. Advances in cognitive neuroscience have further highlighted its complexity and clinical relevance, demonstrating that impulsive behavior is associated with distinct psychological and neural mechanisms and plays a central role in disorders such as attention-deficit/hyperactivity disorder (ADHD), substance-use disorders, pathological gambling, and affective disorders (Dalley and Robbins, 2017).

In their attempt to conceptualize and measure impulsivity as a multifaceted construct, Whiteside and Lynam (2001) devised the four facets of the Urgency-Premeditation-Perseverance-Sensation Impulsive Behavior Scale (UPPS), establishing four distinct components of impulsive behavior: urgency, premeditation, perseverance, and sensation-seeking. Impulsivity reflects multiple self-regulatory processes that differ in their affective and cognitive underpinnings (Carver and Johnson, 2018). Urgency items tap rash emotion-based actions and thus are considered metrics of emotional impulsivity—whereas lack of premeditation and lack of perseverance items tap effortful control and top-down driven cognitive impulsivity (Gomez and Watson, 2023). Neuroimaging studies support this distinction, linking urgency to affective networks and premeditation and perseverance to prefrontal control systems (Golchert et al., 2017).

In the search for underlying neurobiological mechanisms of the foregoing, the neuroendocrine system has been investigated with regard to impulsivity facets. Given that hormones are among the major physiological regulators of brain development and maintenance of homeostasis, neuroendocrine-system dysfunction may result in the elevation of impulsivity (Takahashi, 2004; Aluja et al., 2024). Gonadal hormones are signaling molecules comprised of androgens, estrogens, and progestins. These hormones are produced primarily by the gonads (ovaries in females and testes in males) and also, in both sexes, by the adrenal glands, acting along the hypothalamic-pituitary-gonadal (HPG) axis. Gonadal hormones affect multiple physiological systems including the central nervous system (Coates and Herbert, 2008; Halpern, 2012). Extensive research indicates that gonadal hormones have both organizational and activational effects across the lifespan (Coates and Herbert, 2008). Testosterone (T) has been associated with increased aggression (Archer, 2006) and sensation-seeking (Roberti, 2004) and estradiol (E) and progesterone (P) have been studied with regard to risk-taking. The findings, however, are inconsistent (e.g., Zethraeus et al., 2009; Derntl et al., 2014), remain mixed and do not provide a consistent picture of how gonadal hormones relate to specific facets of impulsivity.

Recent studies have begun to examine the relationship between gonadal hormones and specific impulsivity facets. Martel et al. (2025), for example, found that lack of premeditation moderates inattentive symptoms between midfollicular (low E levels) and midluteal (moderate–high E levels) phases. At high levels of lack of premeditation, inattentive symptom scores are higher in the midfollicular phase than in the midluteal phase. Diekhof (2015) examined the role of estradiol levels in another aspect of impulsivity, reward acquisition, and found that women acted more impulsively at the early stage (lower E levels) than in the late follicular phase (higher E levels). As for the association between T and impulsivity facets, Gomaa et al. (2023), comparing *inter alia* impulsivity between women with polycystic ovary syndrome (PCOS) and women with infertility due to causes other than PCOS, found that the PCOS group exhibited higher levels of urgency and sensation-seeking levels. They suggested that the increase in these impulsivity facets may be attributed to higher androgen levels in patients with PCOS. Recently, Wu et al. (2020) showed that administering a single-dose T increased impulsivity in an intertemporal-choice task in healthy young men. Taken together, these findings suggest that gonadal hormones are associated with several facets of impulsivity. The considerable variability across the studies, however, indicates that these associations are likely to depend on additional biological and psychological factors.

Accumulating evidence suggests that the behavioral effects of gonadal hormones are context-dependent rather than uniform (Rubinow and Schmidt, 2002). Accordingly, hormonal influences may vary as a function of concurrent internal contexts. Two such contexts appear particularly relevant to impulsivity: the neuroendocrine state (e.g., cortisol) and the affective state (e.g., anxiety). We first consider the neuroendocrine context.

Cortisol (C), is the primary human glucocorticoid and the end-product of the hypothalamic-pituitary-adrenal (HPA) axis (Coates and Herbert, 2008). Elevated cortisol levels are associated with stress and avoidance behaviors (e.g., Dickerson and Kemeny, 2004), whereas lower levels are linked to relaxation and approach tendencies (Terburg et al., 2009). Several studies investigate the relationship between C levels and impulsivity. Feilhauer et al. (2013), for example, found a negative association between C levels and impulsivity among male adolescents. In another study including both male and female adolescents, C levels were found negatively associated with impulse control among males but not among females (Poustka et al., 2010). Using experimental hydrocortisone administration, Kluen et al. (2017) demonstrated that acute cortisol elevation increased risk-taking behavior in men but not in women. Taken together, the inconsistent sex-dependent findings imply that impulsivity may be better understood within a framework that accounts for interactions among multiple hormonal systems. One such framework is the *dual-hormone hypothesis* (DHH), which posits that the behavioral effects of T on social-dominance behaviors are contingent upon C levels (Dabbs et al., 1991; Popma et al., 2007; Mehta and Josephs, 2010). According to the DHH, the behavioral effects of T are contingent upon concurrent C levels, highlighting the functional interplay between the HPA and HPG axes. This cross-talk between the adrenal and the gonadal systems is proposed to influence social-dominance–related behaviors (Mehta et al., 2015). As for impulsivity, Singh (2021) showed that C and T jointly modulated risk-taking behavior using the IOWA Gambling Task (IGT) measuring motor impulsivity. Cortisol regulation improved decision-making toward safe rewards. Recently, Borraz-Leon et al. (2023), examining the relationship among T, C, and impulsivity in response to psychosocial stress, found that impulsivity was associated with C reactivity to psychosocial stress, whereas associations with T reactivity were observed only among women. These findings underscore the complex and sex-dependent interplay between impulsivity and endocrine responses to stress.

The involvement of additional gonadal hormones in impulsivity calls for their examination within the DHH framework. Tackett et al. (2015) extended this framework beyond T and C by examining the role of E in adolescents' externalizing behaviors. Their findings indicate that E is an important hormonal target, further supporting the notion of cross-talk between the HPA and HPG axes. Although E is commonly identified as a female hormone, it is also produced in males through the aromatization of circulating T (de Ronde et al., 2003). Consistent with the DHH, E was positively associated with externalizing behavior, but only at low C levels.

Although the dual-hormone hypothesis was originally proposed to explain social-dominance behaviors, its emphasis on interactions between the HPA and HPG axes provides a useful framework for understanding how gonadal hormones may influence impulsivity within a neuroendocrine context. Such interactions are likely to be expressed through neural systems involved in cognitive control and behavioral regulation. Given the established cross-talk between the HPA and HPG axes (Viau, 2002), variability in stress load may alter the functional expression of gonadal-hormone–behavior associations. Converging evidence indicates that elevated cortisol compromises prefrontal efficiency and shifts behavior toward less deliberative responding (Arnsten, 2009; Eysenck et al., 2007). Because regulatory facets of impulsivity rely heavily on prefrontal cognitive-control processes, cortisol may influence the extent to which gonadal hormones are associated with these behavioral tendencies. Estradiol has been implicated in the modulation of prefrontal cognitive-control processes, partly through its effect on dopaminergic signaling and functional dynamics within frontostriatal networks (Jacobs and D'Esposito, 2011; Hampson, 2018). Testosterone, while more frequently examined in relation to approach motivation and dominance-related behaviors, has also been shown to interact with neural systems that support goal-directed action under specific contextual conditions (Mehta and Josephs, 2010). Collectively, this evidence supports the view that neuroendocrine context constitutes an important condition under which associations between gonadal hormones and impulsivity are expressed.

Beyond the neuroendocrine milieu, another important internal context through which gonadal hormones may influence impulsivity is the individual's affective state. In particular, anxiety has been proposed as a key affective context that may modify the relation between gonadal hormones and social dominance, aggression, and risk-taking behaviors. Norman et al. (2015), for example, examined across two studies the moderating role of trait anxiety in the relationship between T responses to competition (through saliva samples) and aggressive behavior. Their findings indicate a positive association between T responses to competition and aggression, but only among men with low levels of trait anxiety. In a similar vein, Ziomkiewicz et al. (2015) measured self-assessed social dominance and trait anxiety of young female participants (mean age 26.3) and took T, E, and C serum concentrations during the follicular phase of their menstrual cycle. They showed that E and the E–T ratio are negatively associated with social dominance and that anxiety levels moderated these associations. That is, lower E and E–T ratio levels are associated with higher social dominance among moderately and highly anxious participants but not among low-anxiety participants. To the best of our knowledge, the moderating role of anxiety has not yet been studied in the relation between hormones and impulsivity. Studies identify anxiety as a strong predictor of impulsivity (e.g., Moustafa et al., 2017). Yu et al. (2020), for example, investigating the relationship between anxiety and various impulsivity components, showed that anxiety is positively associated with attention impulsivity, motor impulsivity, and non-planning impulsivity.

In sum, despite growing interest in hormonal influences on impulsive behavior, little is known about how gonadal hormones relate to specific facets of impulsivity. Moreover, accumulating evidence suggests that hormonal influences on behavior are often context-dependent and may vary as a function of concurrent neuroendocrine and affective states. In particular, activity of the HPA axis, indexed by cortisol, has been shown to alter prefrontal regulatory processes and may therefore qualify associations between gonadal hormones and impulsivity-related behavior. Similarly, momentary anxious states may impose additional regulatory demands and thereby shape the behavioral expression of hormonal influences.

Accordingly, in this study we asked whether the associations between gonadal hormones (*T* and *E*) and impulsivity facets vary as a function of internal regulatory states. Specifically, we tested whether cortisol levels and state anxiety moderate the associations between gonadal hormones and the distinct dimensions of impulsivity as assessed within the UPPS framework. Because previous evidence linking gonadal hormones to specific impulsivity facets is scarce and often inconsistent, we formulated no directional hypotheses regarding individual UPPS facets.

To address the questions asked above, the study examined associations between gonadal hormones and impulsivity facets in a mixed sample of men and women in different hormonal states. Specifically, the sample included men, women using oral contraceptives, and naturally cycling women in the mid-luteal phase. This design made it possible to examine hormonal associations across distinct endocrine contexts. Because P levels are substantially elevated during the luteal phase and may contribute to neurobehavioral functioning, P was measured and included as a covariate to account for its potential influence.

Our hypotheses were the following:

H1: Cortisol levels moderate the associations between gonadal hormones and impulsivity facets.

H2: Anxiety moderates the associations between gonadal hormones and impulsivity facets.

## 2 Methods

### 2.1 Participants

The participants were 197 undergraduate students (66 men, mean age 26.14 ± 3.08 years). Among the women, 68 used oral contraceptives (the oral-contraceptives group, OC; mean age 23.48 ± 1.80 years); the remaining 63 did not use oral contraceptives and were tested during the mid-luteal phase of their menstrual cycle (luteal phase group, LP; mean age 24.06 ± 2.62 years). Participants were recruited through advertisements posted on college notice boards. Inclusion criteria for all participants included good general health, absence of serious medical, endocrine, or psychiatric conditions, non-smoking status, and no self-reported diagnosis of attention-deficit/hyperactivity disorder (ADHD) or learning disabilities. All male participants met the inclusion criteria. Female participants underwent an additional pre-screening procedure to ensure eligibility. Women in the OC group were tested during the active pill phase and were using oral contraceptives containing 25 mg of estrogen (ethinylestradiol) and 75 mg of progestin (gestodene). These dosages are considered moderate, are commonly prescribed, and are characterized by antiandrogenic properties (Montoya and Bos, 2017). All women in this group had been using oral contraceptives for at least 1 year. Women in the LP group had not taken oral contraceptives for at least 6 months before participation, reported a regular menstrual cycle, and were neither pregnant nor lactating. These participants were monitored for at least 3 months before the study to confirm regularity and were scheduled for laboratory testing on the 21st day of their cycle, a calculation based on the onset of their most recent menstruation (Rossi and Rossi, 1977). Testing of women in their mid-luteal phase complied with established recommendations from previous studies (e.g., Dreyer et al., 2022) because women in this phase exhibit basal cortisol levels more comparable with those of men, reducing menstrual-cycle-related variability in cortisol while allowing P to be measured and statistically controlled.

Statistical power was estimated a priori using G^*^Power (Faul et al., 2009). The minimum total sample size required to detect a small-to-moderate effect in the planned moderation analyses, assuming α = 0.05 and 80% power, was estimated at 164 participants (177 for models including the multicategorical endocrine group variable). To account for potential missing data and exclusions, we aimed to recruit approximately 200 participants. Recruitment was planned to achieve a roughly balanced distribution across the three endocrine groups.

### 2.2 Measures

The study used self-report questionnaires to measure the following:

*Personal details:* sociodemographic information including age, gender, and study discipline.

*Impulsivity:* measured using the validated UPPS behavior scale designed by Whiteside and Lynam (2001). The UPPS measures four distinct personality pathways to impulsive behavior: Premeditation (lack of), Urgency, Sensation Seeking, and Perseverance (lack of). The UPPS is a 45-item inventory with a Likert-type format ranging from one to four. Internal reliabilities (Cronbach's alpha) in the present sample were 0.93, 0.86, 0.79, and 0.81 for Premeditation (lack of), Urgency, Sensation Seeking, and Perseverance (lack of), respectively.

*Anxiety:* assessed using the State-Trait Inventory (STAI-S) (Spielberger, 1983), a widely used and well-validated measure that assesses the extent of current feeling of anxiety. Its 20 items are scored from 1 (not at all) to 4 (very much so). The STAI has demonstrated very good internal consistency and good item characteristics (Ilardi et al., 2021) and previous studies provide normative data for various age groups and countries (e.g., Potvin et al., 2011; Bergua et al., 2012; Ilardi et al., 2021). We summed and averaged the items to create an index of state anxiety (Cronbach's alpha = 0.93).

#### 2.2.1 Biomarker measures

To test endogenous hormone levels, *C, E*, and *T* levels were measured. P concentrations were measured and included as a covariate in all analyses to account for hormonal variation associated with the luteal phase. To control for diurnal-rhythm changes in C and T, participants were tested between 12:00 and 18:00. They were asked to refrain from eating, drinking (except water), and smoking for at least 1 h before arriving at the laboratory. Ahead of saliva sampling, participants were instructed to chew on a piece of parafilm for several seconds to increase saliva secretion. They then deposited 2.5 ml of saliva in a SaliCap sampling vial (IBL International GMBH, Hamburg, Germany). Saliva samples were stored at −20 °C from collection until the laboratory tests were performed. For each biochemical analyte, tests were performed using commercial CE-IVD-approved ELISA kits: cortisol saliva ELISA (mean intra-assay CV% = 4.7, mean inter-assay CV% = 10.6, assay sensitivity = 0.14 nmol/L), testosterone saliva ELISA (mean intra-assay CV% = 5.57, mean inter-assay CV% = 5.7, assay sensitivity = 8.6 pg/mL), 17 beta estradiol saliva ELISA (mean intra-assay CV% = 8.8, mean inter-assay CV% =11.8, assay sensitivity = 2.1 pg/mL; all from IBL International GMBH, Hamburg, Germany). All tests were performed in an SQII ELISA processor (AESKU Systems, Wendelsheim, Germany) at the endocrinology laboratory of Emek Medical Center, an ISO 9001 (2015 version) certified and JCI-accredited facility. All analytical kits used in the study were previously validated in the laboratory in accordance with good laboratory practice standards. A calibration curve using standard duplicates was performed for each analyte in every run.

### 2.3 Procedure

The study was approved by the institutional ethical review board (IRB) of the Max Stern Yezreel Valley College. After giving their fully informed consent, participants completed a brief demographic questionnaire. All participants were then tested for biomarkers, after which they completed the questionnaires.

### 2.4 Data analysis

All statistical analyses were conducted using IBM SPSS Statistics (Version 28). To reduce skew, T, E, and C values were log-transformed prior to analysis. E and T were subsequently standardized (*z*-scores) within each group (men, oral-contraceptive users, and naturally cycling women in the luteal phase) following procedures commonly used in studies including mixed-sex samples (e.g., Tackett et al., 2015). Anxiety scores were also standardized to facilitate interpretation of interaction effects. Group differences in hormone levels were examined using one-way analyses of variance (ANOVA) followed by Scheffé *post-hoc* tests for pairwise comparisons among the three groups. A Bonferroni correction was applied to the correlation matrix (Table 1) to account for multiple comparisons. Significance was set at *p* < 0.002. To test the moderation hypotheses, moderated regression analyses were conducted. P levels were included as a covariate in all regression models in order to account for potential effects of luteal-phase hormonal variability. Because these models were theory-driven, additional corrections for multiple testing were not applied. All analyses were performed on the full sample using group-standardized hormone values. Significant interaction effects were probed using the procedures described by Aiken et al. (1991). Specifically, simple slope analyses were conducted to estimate the association between the hormonal predictor and impulsivity facets at one standard deviation below (low) and above (high) the mean levels of the moderator (cortisol or anxiety).

**Table 1:** Correlations between hormone levels (T, E, P, and C), anxiety, and impulsivity sub-scales.

Variable	1	2	3	4	5	6	7
1. C							
2. T	−0.11						
3. E	**0.52** ^***^	0.10					
4. Anxiety	−0.06	0.03	−0.08				
5. Premeditation	**0.39** ^***^	**−0.26** ^***^	**0.41** ^***^	0.06			
6. Urgency	**0.26** ^***^	−0.10	0.12	0.41^***^	0.04		
7. Sensation-seeking	0.22^**^	0.13	**0.25** ^***^	−0.18	0.07	0.08	
8. Perseverance	0.21^**^	−0.13	**0.31** ^***^	**−0.35** ^***^	**0.76** ^***^	−0.16^*^	0.09

^*^p < 0.05, ^**^p < 0.01, and ^***^p < 0.001.

C, cortisol; T, testosterone; E, estradiol.

Values in boldface represent the only correlation that remained statistically significant after application of Bonferroni correction for multiple comparisons (α = 0.05/28 = 0.002).

## 3 Results

### 3.1 Hormone levels

Table 2 presents group-level hormone differences. The one-way ANOVA revealed a significant difference among the groups in T, E, and P levels. The Scheffé's *post-hoc* analysis demonstrated significant differences between men and both groups of women in T levels, men having higher T levels than women. In addition, the Scheffé's *post-hoc* analysis demonstrated significant differences between the LP group and both men and the OC group in E and P levels, LP women having higher E and P levels than OC women and men. No significant differences in C levels were found (Table 2).

**Table 2:** Means (SEM) and *F* and *P*-values for sex differences in hormones (T, E, P, and C).

Hormone	Men (*N* = 66)	OC (*N* = 68)	LP (*N* = 63)	*F*
Cortisol	6.84 (1.20)	6.99 (1.07)	7.06 (1.30)	0.01
Testosterone	122.46 (8.37)	25.96 (2.07)	39.16 (3.42)	96.00^***^
Estradiol	2.96 (0.13)	2.60 (0.08)	3.58 (0.14)	17.35^***^
Progesterone	48.82 (3.93)	36.05 (3.09)	136.06 (18.47)	26.76^***^

^***^p < 0.001.

SEM, standard error of the mean; OC, oral contraceptives; LP, luteal phase.

### 3.2 Hormones, anxiety, and impulsivity facets

Table 1 presents correlations among hormone levels, anxiety, and impulsivity facets. E levels were positively associated with sensation-seeking and with lack of premeditation and perseverance. T levels were negatively associated with lack of premeditation. C levels were positively associated with urgency and lack of premeditation. Supplementary Table S1 presents correlations among study variables for each endocrine group.

Table 3 presents a series of multiple regression analyses with premeditation, urgency, sensation-seeking, and perseverance as dependent variables and with T, C, E, T × C, and E × C interactions. There was significant E × C interaction on premeditation. Decomposing this interaction (Hayes, 2013), we uncovered a positive association between E and premeditation when C was low (*b* = 0.28, se = 0.07, *t* = 3.99, and *p* < 0.001) but a negative association between E and premeditation when C was high (*b* = −0.31, se = 0.10, *t* = −3.02, and *p* < 0.01). In addition, there was a significant E × C interaction on perseverance. Decomposing this interaction, we found a positive association between E and perseverance when C was low (*b* = 0.14, se = 0.04, *t* = 3.10, and *p* < 0.01) but a non-significant negative association between E and perseverance when C was high (*b* = −0.11, se = 0.07, *t* = −1.63, and *p* > 0.05).

**Table 3:** The moderating role of C in the relation between gonadal hormones and impulsivity sub-scales.

Predictors	*B*	se	*t*	*p*	[95% CI]
Premeditation					
T	−0.17	0.06	−2.69	0.008	[−0.30, −0.05]
C	0.25	0.06	4.49	0.000	[0.14, 0.36]
T × C	−0.02	0.07	−0.33	0.741	[−0.15, 0.11]
E	−0.02	0.07	−0.24	0.812	[−0.15. 0.12]
C	0.25	0.06	4.14	0.0001	[0.13, 0.37]
E × C	−0.30	0.06	−5.20	0.000	[−0.42, −0.19]
Urgency					
T	−0.04	0.05	−0.71	0.479	[−0.15, 0.07]
C	0.17	0.05	3.50	0.0006	[0.07, 0.26]
T × C	−0.01	0.06	−0.02	0.809	[−0.13, 0.10]
E	0.01	0.06	0.22	0.824	[−0.11. 0.13]
C	0.16	0.05	2.93	0.004	[0.05, 0.27]
E × C	0.01	0.05	0.28	0.783	[−0.09, 0.12]
Sensation-seeking					
T	0.15	0.06	2.56	0.011	[0.03, 0.26]
C	0.18	0.05	3.77	0.0002	[0.09, 0.28]
T × C	0.01	0.06	0.23	0.819	[−0.10, 0.13]
E	0.11	0.06	1.74	0.084	[−0.01. 0.23]
C	0.13	0.07	2.33	0.021	[0.02, 0.24]
E × C	−0.01	0.05	−0.23	0.819	[−0.12, 0.09]
Perseverance					
T	−0.02	0.04	−0.62	0.539	[−0.10, 0.05]
C	0.05	0.03	1.48	0.140	[−0.02, 0.12]
T × C	0.02	0.04	0.54	0.589	[−0.06, 0.10]
E	0.02	0.04	0.37	0.715	[−0.07, 0.10]
C	0.04	0.04	1.08	0.280	[−0.03, 0.12]
E × C	−0.13	0.04	−3.39	0.001	[−0.20, −0.05]

B indicates unstandardized regression coefficients. CI, confidence interval; T, testosterone; C, cortisol; E, estradiol.

Table 4 presents a series of multiple regression analyses with premeditation, urgency, sensation-seeking, and perseverance as dependent variables and with T, E, anxiety, T × anxiety, and E × anxiety interactions. There was a significant E × anxiety interaction on premeditation. Simple slope analyses indicated that the negative association between E and premeditation were more pronounced at higher anxiety levels, although the simple slope did not attain conventional significance (*b* = −0.16, se = 0.10, *t* = −1.73, and *p* = 0.088). In addition, a significant T × anxiety interaction with perseverance was found. Decomposing this interaction, we detected a positive association between T and perseverance when anxiety was high (*b* = 0.22, se = 0.07, *t* = 3.15, and *p* < 0.01) but a non-significant negative association between T and perseverance when anxiety was low (*b* = −0.08, se = 0.06, *t* = −1.28, and *p* > 0.05). Furthermore, there was a significant E × anxiety interaction on perseverance. Decomposing this interaction, we discovered a positive association between E and perseverance when anxiety was low (*b* = 0.19, se = 0.08, *t* = 2.51, and *p* < 0.05) but a negative association between E and perseverance when anxiety was high (*b* = −0.16, se = 0.07, *t* = −2.12, and *p* < 0.05).

**Table 4:** The moderating role of anxiety in the relation between gonadal hormones and impulsivity sub-scales.

Predictors	*B*	se	*t*	*p*	[95% CI]
Premeditation					
T	−0.00	0.05	−0.06	0.949	[−0.11, 0.10]
Anxiety	0.02	0.05	0.48	0.627	[−0.07. 0.12]
T × anxiety	0.10	0.06	1.62	0.110	[−0.02, 0.22]
E	−0.02	0.07	−0.22	0.827	[−0.15. 0.12]
Anxiety	0.02	0.05	0.51	0.613	[−0.07, 0.12]
E × anxiety	−0.15	0.07	−2.22	0.029	[−0.28, −0.02]
Urgency					
T	−0.07	0.08	−0.93	0.354	[−0.22, 0.08]
Anxiety	0.30	0.07	4.30	0.000	[0.16, 0.44]
T × anxiety	−0.09	0.09	−1.04	0.303	[−0.26, 0.08]
E	0.04	0.09	0.43	0.668	[−0.15. 0.23]
Anxiety	0.30	0.07	4.22	0.0001	[0.15, 0.44]
E × anxiety	−0.03	0.10	−0.31	0.759	[−0.22, 0.16]
Sensation-seeking					
T	0.27	0.08	3.26	0.002	[0.10, 0.44]
Anxiety	−0.13	0.08	−1.70	0.092	[−0.29, 0.02]
T × anxiety	−0.06	0.10	−0.66	0.508	[−0.25, 0.13]
E	0.16	0.11	1.42	0.159	[−0.06. 0.37]
Anxiety	−0.12	0.08	−1.48	0.143	[−0.29, 0.04]
E × anxiety	0.02	0.13	0.25	0.802	[−0.22, 0.28]
Perseverance					
T	0.07	0.04	1.59	0.116	[−0.02, 0.15]
Anxiety	−0.16	0.04	−3.97	0.0002	[−0.24, −0.08]
T × anxiety	0.15	0.05	3.02	0.003	[0.05, 0.24]
E	0.01	0.05	0.25	0.809	[−0.09, 0.12]
Anxiety	−0.15	0.04	−3.84	0.0002	[−0.23, −0.07]
E × anxiety	−0.18	0.06	−3.23	0.002	[−0.29, −0.08]

B indicates unstandardized regression coefficients. CI, confidence interval; T, testosterone; E, estradiol.

To examine whether anxiety and cortisol jointly qualified hormonal associations with impulsivity, a three-way interaction model (T or E × anxiety × cortisol) was tested. None of the three-way interaction terms was significant (all *p*s > 0.05).

Additional analyses were conducted to test the robustness of the findings across endocrine groups. First, moderated moderation analyses revealed that none of the hormone × moderator × endocrine group interactions reached statistical significance, providing no evidence that the observed moderation effects differed across endocrine groups (Supplementary Tables S2, S3). Second, all moderation models were re-estimated with two dummy-coded covariates used to adjust for endocrine-group membership. The pattern of significant hormone × moderator interactions remained unchanged, supporting the robustness of the primary findings (Supplementary Tables S4, S5).

## 4 Discussion

This study asked whether associations between gonadal hormones and distinct facets of impulsivity are contingent upon internal neuroendocrine and affective states. Overall, the findings indicate that hormonal influences on impulsivity are not uniform but emerge under specific contextual conditions. Interactions between E and C as well as between E and state anxiety were observed for the impulsivity facets of premeditation and perseverance. In addition, the association between T and perseverance varied as a function of anxiety levels. Notably, these effects were specific to regulatory facets of impulsivity and were not observed for urgency or sensation-seeking. Together, these findings suggest that links between gonadal hormones and impulsive behavior may become evident primarily when concurrent neuroendocrine and affective contexts are considered.

As expected and in accordance with previous studies (e.g., Merz, 2017), the three endocrine groups differed in their gonadal hormone profiles. Men showed higher T concentrations than did both groups of women, whereas LP women showed higher levels of ovarian hormones. In contrast, C levels did not differ significantly across the groups.

Our first hypothesis, that C levels would moderate the associations between gonadal hormones and impulsivity facets, was partly supported. Specifically, C levels moderated the associations between E and the impulsivity facets of lack of premeditation and lack of perseverance. These facets are generally considered regulatory components of impulsivity that rely on cognitive control processes (Carver and Johnson, 2018). The findings suggest that associations between E and impulsive tendencies may be contingent upon concurrent activity of the HPA axis. At relatively low C levels, higher E levels were associated with greater impulsive tendencies, whereas this association was attenuated or reversed when C levels were higher. Such context-dependent hormonal effects are consistent with previous research that demonstrates cross-talk between the HPA and HPG axes. For example, Tackett et al. (2015) reported that E was positively associated with externalizing behavior only at low C levels, a pattern broadly consistent with predictions derived from the dual-hormone framework.

Beyond a neuroendocrine context indexed by cortisol levels, affective states such as anxiety may also influence the way gonadal hormones are expressed in behavior, particularly in domains related to cognitive control and impulsivity. Consistent with this notion, our second hypothesis—that anxiety would moderate the associations between gonadal hormones and impulsivity facets—was partly supported. Anxiety moderated the associations between E and T and the impulsivity facets of lack of premeditation and lack of perseverance. At higher levels of anxiety, E was associated with lower levels of lack of premeditation and lack of perseverance, suggesting reduced impulsive tendencies in these domains. In contrast, T showed an opposite pattern, such that higher T levels were associated with greater lack of perseverance at elevated anxiety levels. This differential pattern of hormonal associations with cognitive-related facets of impulsivity is broadly consistent with previous findings indicating that the behavioral effects of gonadal hormones may depend on affective context. For example, Ziomkiewicz et al. (2015) reported that lower E levels and lower E–T ratios were associated with higher social dominance among moderately and highly anxious individuals but not among individuals with low anxiety levels.

Our findings extend previous work suggesting that hormonal influences on behavior vary across contextual conditions. Vermeersch et al. (2008) reported that E is associated with aggression and risk-taking among adolescent girls and found the association stronger among girls whose peers engaged in higher levels of risk-taking. Similarly, Rowe et al. (2004) suggested that the behavioral effects of *T* and *E* on risk-taking may differ across social contexts. Extending this perspective, our findings indicate that hormonal associations with impulsivity may depend not only on social environments but also on internal neuroendocrine and affective contexts.

Importantly, our findings are specific to the facets of premeditation and perseverance. Contemporary models of impulsivity distinguish between processes driven by emotional reactivity and those related to cognitive control. Consistent with this distinction, scholars have described emotional (or reflexive) and cognitive (or reflective) forms of impulsivity (Carver and Johnson, 2018). Emotional impulsivity reflects rapid, automatic responses that emerge from heightened emotional states and is typically driven by bottom-up processes (Whiteside and Lynam, 2001), whereas cognitive impulsivity reflects impulsive response stemming from limitations in cognitive control and is associated with top-down regulatory mechanisms (Carver and Johnson, 2018).

Neuroimaging findings support this distinction. Golchert et al. (2017), using resting-state functional connectivity of the anterior cingulate cortex (ACC), found an association between urgency and networks implicated in affective processing, as against an association between lack of premeditation and lack of perseverance with connectivity in regions supportive of cognitive control and attentional regulation, including the dorsolateral prefrontal cortex and inferior frontal gyrus.

Within this framework, the observed hormonal associations may reflect modulation of prefrontal regulatory processes. Gonadal hormones are known neuromodulators of higher-order cognitive functions and prefrontal cortex (PFC) activity (e.g., Taxier et al., 2020). Previous findings suggest that estradiol regulates PFC function through modulation of specific neurochemical systems (e.g., Aubele and Kritzer, 2011; Jacobs and D'Esposito, 2011). (Jacobs and D'Esposito 2011), for example, demonstrated that estradiol effects on working memory—a PFC-dependent cognitive function—are influenced by baseline dopamine signaling in the PFC. The moderated associations observed in our study between estradiol and the facets of premeditation and perseverance may therefore reflect hormonal modulation of PFC-dependent regulatory processes.

An important finding was the absence of significant three-way interactions involving endocrine groups. This suggests that the observed hormone–impulsivity associations moderated by cortisol or anxiety did not significantly differ across the endocrine groups included in this study. Given the relatively modest sample sizes within each group, however, subtle group-specific patterns cannot be ruled out. Future studies with larger samples should further examine whether distinct endocrine profiles are associated with different patterns of hormone–impulsivity associations across men, naturally cycling women, and women using oral contraceptives.

Several limitations of the present study should be acknowledged. First, the cross-sectional design precludes causal inferences about the directionality of the observed associations. Longitudinal or experimental approaches would allow stronger conclusions regarding the mechanisms through which hormonal and affective contexts interact to shape impulsive behavior. Second, we assessed hormonal levels at a single time point, possibly ruling out full capture of dynamic fluctuations in endocrine activity. Future studies would benefit from repeated hormonal measurements across multiple time points. Third, naturally cycling women were assessed during the mid-luteal phase, which is characterized by relatively elevated E and P levels. Although this approach allowed partial control over hormonal status, it does not capture hormonal changes across other phases of the menstrual cycle—particularly the peri-ovulatory phase, in which estradiol peaks. Fourth, we assessed impulsivity facets using self-report measures rather than behavioral tasks that directly capture cognitive control processes. Finally, anxiety was assessed at a single time point and may not fully reflect fluctuations in affective states that may influence hormonal–behavioral associations.

In conclusion, the present findings suggest that associations between gonadal hormones and impulsivity may vary as a function of concurrent neuroendocrine and affective states. These patterns appear more evident in regulatory facets of impulsivity, highlighting the importance of distinguishing between functional components of impulsive behavior. By pointing to potential interactions between HPA and HPG activity in relation to behavioral tendencies, this study supports a biobehavioral perspective in which hormonal-behavioral associations are context-sensitive. Future research using experimental and longitudinal designs will be important for clarifying the nature and direction of these relationships.

## Data Availability

The raw data supporting the conclusions of this article will be made available by the authors, without undue reservation.
