# Supplementary material for: Gonadal hormones and impulsivity facets: moderating roles of cortisol and anxiety
Source: Front Psychol. 2026 Sep 3;17:1871253. doi: 10.3389/fpsyg.2026.1871253 (PMC13581836; doi:10.3389/fpsyg.2026.1871253)
Supplement: Supplementary file 1 [file Table_1.docx]

Table S1. Correlations between hormone levels (T, E, P, C), anxiety, and impulsivity sub-scales in each endocrine group

|  | **1** | | | **2** | | | **3** | | | **4** | | | **5** | | | **6** | | | **7** | | |
| --- | --- | --- | --- | --- | --- | --- | --- | --- | --- | --- | --- | --- | --- | --- | --- | --- | --- | --- | --- | --- | --- |
|  | **M** | **OC** | **LP** | **M** | **OC** | **LP** | **M** | **OC** | **LP** | **M** | **OC** | **LP** | **M** | **OC** | **LP** | **M** | **OC** | **LP** | **M** | **OC** | **LP** |
| **1. C** |  |  |  |  |  |  |  |  |  |  |  |  |  |  |  |  |  |  |  |  |  |
| **2. T** | -0.03 | -0.15 | -0.15 |  |  |  |  |  |  |  |  |  |  |  |  |  |  |  |  |  |  |
| **3. E** | **0.63***** | **0.42***** | **0.51***** | 0.29* | 0,10 | -0.07 |  |  |  |  |  |  |  |  |  |  |  |  |  |  |  |
| **4. Anxiety** | -0.06 | 0.21 | -0.11 | 0.15 | 0.35** | -0.11 | -0.25* | 0.09 | -0.18 |  |  |  |  |  |  |  |  |  |  |  |  |
| **5. Premeditation** | 0.37** | **0.43***** | 0.37** | -0.17 | **-0.41***** | -0.19 | 0.42*** | 0.25* | **0.56***** | **-0.49***** | **-0.39***** | -0.42*** |  |  |  |  |  |  |  |  |  |
| **6. Urgency** | 0.26* | 0.30* | 0.19 | -0.11 | -0.16 | -0.02 | 0.11 | 0.09 | 0.17 | 0.38** | 0.28* | 0.33** | 0.07 | 0.05 | -0.03 |  |  |  |  |  |  |
| **7. Sensation- seeking** | 0.14 | 0.22 | 0.40** | 0.21 | 0.03 | 0.17 | 0.31* | 0.14 | 0.32* | -0.16 | 0,05 | -0.08 | 0.15 | -0.00 | 0.10 | 0.08 | 0.13 | 0.17 |  |  |  |
| **8. Perseverance** | 0.31* | 0.19 | 0.14 | -0.10 | -0.31** | 0.05 | 0.40** | 0.19 | 0.37** | **0.63***** | **-0.48***** | **-0.53***** | **0.75***** | **0.80***** | **0.76***** | -0.15 | -0.08 | -0.26 | 0.24 | -0.14 | 0.18 |

Note: * *p* < .05, ** *p* < .01, *** *p* < .001

Abbreviations: C: cortisol; T: testosterone; E: estradiol; LP = luteal-phase women; OC = oral-contraceptive users.

Values in boldface represent the only correlation that remained statistically significant after application of Bonferroni correction for multiple comparisons (α = .05 / 84 = .0006).

Table S2. Three-way hormone × cortisol × endocrine group interactions predicting impulsivity subscales

|  | *Comparison* | *B* | *se* | *p* | *[95% CI]* |
| --- | --- | --- | --- | --- | --- |
| Premeditation |  |  |  |  |  |
| T × C × group | OC vs. M | 0.06 | 0.16 | .725 | [-0.26, 0.37] |
|  | LP vs. M | 0.28 | 0.17 | .100 | [-0.05, 0.62] |
| E × C × group | OC vs. M | 0.20 | 0.14 | .154 | [-0.06. 0.47] |
|  | LP vs. M | -0.09 | 0.16 | .573 | [-0.40, 0.22] |
| Urgency |  |  |  |  |  |
| T × C × group | OC vs. M | 0.06 | 0.13 | .624 | [-0.20, 0.32] |
|  | LP vs. M | -0.22 | 0.14 | .128 | [-0.50, 0.06] |
| E × C × group | OC vs. M | 0.15 | 0.12 | .225 | [-0.09, 0.40] |
|  | LP vs. M | -0.09 | 0.14 | .532 | [-0.36, 0.19] |
| Sensation-seeking |  |  |  |  |  |
| T × C × group | OC vs. M | -0.08 | 0.14 | .571 | [-0.35, 0.19] |
|  | LP vs. M | 0.06 | 0.15 | .674 | [-0.23, 0.35] |
| E × C × group | OC vs. M | 0.04 | 0.13 | .782 | [-0.22, 0.29] |
|  | LP vs. M | -0.00 | 0.14 | .337 | [-0.29, 0.28] |
| Perseverance |  |  |  |  |  |
| T × C × group | OC vs. M | -0.04 | 0.10 | .651 | [-0.24, 0.15] |
|  | LP vs. M | 0.11 | 0.11 | .317 | [-0.10, 0.31] |
| E × C × group | OC vs. M | 0.11 | 0.90 | .219 | [-0.07, 0.29] |
|  | LP vs. M | 0.00 | 0.10 | .987 | [-0.20, 0.20] |

*Note.* Unstandardized regression coefficients are reported. Men served as the reference group. The LP and OC coefficients represent differences in the hormone × moderator interaction relative to men. CI = confidence interval; LP = luteal-phase women; OC = oral-contraceptive users. B indicates unstandardized regression coefficients. T = testosterone. C = cortisol. E = estradiol.

Table S3. Three-way hormone × anxiety × endocrine group interactions predicting impulsivity subscales

|  | *Group comparison* | *B* | *se* | *p* | *[95% CI]* |
| --- | --- | --- | --- | --- | --- |
| Premeditation |  |  |  |  |  |
| T × Anxiety × group | OC vs. M | 0.27 | 0.17 | .116 | [-0.07, 0.60] |
|  | LP vs. M | 0.33 | 0.17 | .053 | [-0.004, 0.66] |
| E × Anxiety × group | OC vs. M | 0.20 | 0.25 | .704 | [-0.40. 0.58] |
|  | LP vs. M | -0.19 | 0.26 | .475 | [-0.71, 0.33] |
| Urgency |  |  |  |  |  |
| T × Anxiety × group | OC vs. M | 0.23 | 0.24 | .353 | [-0.26, 0.71] |
|  | LP vs. M | 0.21 | 0.14 | .353 | [-0.27, 0.69] |
| E × Anxiety × group | OC vs. M | -0.08 | 0.35 | .821 | [-0.78, 0.62] |
|  | LP vs. M | 0.06 | 0.37 | .877 | [-0.68, 0.80] |
| Sensation-seeking |  |  |  |  |  |
| T × Anxiety × group | OC vs. M | -0.19 | 0.27 | .485 | [-0.72, 0.34] |
|  | LP vs. M | -0.19 | 0.27 | .477 | [-0.72, 0.34] |
| E × Anxiety × group | OC vs. M | 0.02 | 0.41 | .961 | [-0.80, 0.84] |
|  | LP vs. M | -0.13 | 0.43 | .769 | [-0.99, 0.74] |
| Perseverance |  |  |  |  |  |
| T × Anxiety × group | OC vs. M | 0.17 | 0.14 | .204 | [-0.10, 0.45] |
|  | LP vs. M | 0.22 | 0.14 | .115 | [-0.06, 0.49] |
| E × Anxiety × group | OC vs. M | -0.03 | 0.20 | .887 | [-0.43, 0.38] |
|  | LP vs. M | -0.22 | 0.21 | .327 | [-0.07, 0.22] |

*Note.* Unstandardized regression coefficients are reported. Men served as the reference group. The LP and OC coefficients represent differences in the hormone × moderator interaction relative to men. CI = confidence interval; LP = luteal-phase women; OC = oral-contraceptive users. B indicates unstandardized regression coefficients. T = testosterone. E = estradiol.

Table S4. The moderating role of C in the relation between gonadal hormones and impulsivity sub-scales controlling for endocrine group

|  | *B* | *se* | *t* | *p* | *[95% CI]* |
| --- | --- | --- | --- | --- | --- |
| Premeditation |  |  |  |  |  |
| T | -0.17 | 0.07 | -2.66 | .009 | [-0.30, -0.04] |
| C | 0.25 | 0.06 | 4.47 | .000 | [0.14, 0.36] |
| T × C | -0.02 | 0.07 | -0.31 | .759 | [-0.15, 0.11] |
| E | -0.02 | 0.07 | -0.24 | .807 | [-0.15. 0.12] |
| C | 0.25 | 0.06 | 4.13 | .000 | [0.13, 0.37] |
| E × C | -0.30 | 0.06 | -5.19 | .000 | [-0.42, -0.19] |
| Urgency |  |  |  |  |  |
| T | -0.03 | 0.05 | -0.58 | .560 | [-0.14, 0.08] |
| C | 0.17 | 0.05 | 3.55 | .001 | [0.07, 0.26] |
| T × C | -0.00 | 0.06 | -0.07 | .942 | [-0.11, 0.11] |
| E | 0.02 | 0.06 | 0.26 | .792 | [-0.10. 0.13] |
| C | 0.16 | 0.05 | 3.00 | .003 | [0.05, 0.27] |
| E × C | 0.02 | 0.05 | 0.45 | .656 | [-0.08, 0.12] |
| Sensation-seeking |  |  |  |  |  |
| T | 0.14 | 0.06 | 2.43 | .016 | [0.03, 0.24] |
| C | 0.18 | 0.05 | 3.86 | .000 | [0.09, 0.28] |
| T × C | 0.00 | 0.06 | 0.02 | .985 | [-0.11, 0.11] |
| E | 0.11 | 0.06 | 1.72 | .009 | [-0.02. 0.23] |
| C | 0.13 | 0.05 | 2.36 | .002 | [0.02, 0.24] |
| E × C | -0.02 | 0.05 | -0.48 | .635 | [-0.13, 0.08] |
| Perseverance |  |  |  |  |  |
| T | -0.03 | 0.04 | -0.65 | .518 | [-0.11, 0.05] |
| C | 0.05 | 0.03 | 1.48 | .141 | [-0.02, 0.12] |
| T × C | 0.02 | 0.04 | 0.49 | .624 | [-0.06, 0.10] |
| E | 0.02 | 0.04 | 0.35 | .727 | [-0.07, 0.10] |
| C | 0.04 | 0.04 | 1.08 | .283 | [-0.03, 0.12] |
| E × C | -0.13 | 0.04 | -3.43 | .0007 | [-0.20, -0.05] |

*Note.* B indicates unstandardized regression coefficients. CI = confidence interval. T = testosterone. C = cortisol. E = estradiol. Endocrine group was entered as two dummy-coded covariates.

Table S5. The moderating role of anxiety in the relation between gonadal hormones and impulsivity sub-scales controlling for endocrine group

|  | *B* | *se* | *t* | *p* | *[95% CI]* |
| --- | --- | --- | --- | --- | --- |
| Premeditation |  |  |  |  |  |
| T | -0.01 | 0.05 | -0.14 | .890 | [-0.12, 0.10] |
| Anxiety | 0.02 | 0.05 | 0.35 | .725 | [-0.12. 0.10] |
| T × anxiety | 0.11 | 0.06 | 1.81 | .074 | [-0.01, 0.24] |
| E | -0.03 | 0.07 | -0.40 | .693 | [-0.16. 0.11] |
| Anxiety | 0.02 | 0.05 | 0.35 | .730 | [-0.08, 0.12] |
| E × anxiety | -0.15 | 0.07 | -2.20 | .030 | [-0.29, -0.01] |
| Urgency |  |  |  |  |  |
| T | -0.06 | 0.08 | -0.85 | .400 | [-0.22, 0.09] |
| Anxiety | 0.29 | 0.07 | 4.04 | .000 | [0.15, 0.43] |
| T × anxiety | -0.10 | 0.09 | -1.10 | .277 | [-0.27, 0.08] |
| E | 0.05 | 0.10 | 0.50 | .620 | [-0.15. 0.24] |
| Anxiety | 0.29 | 0.07 | 3.94 | .000 | [0.14, 0.43] |
| E × anxiety | -0.03 | 0.10 | -0.41 | .685 | [-0.23, 0.15] |
| Sensation-seeking |  |  |  |  |  |
| T | 0.27 | 0.08 | 3.24 | .002 | [0.10, 0.44] |
| Anxiety | -0.10 | 0.08 | -1.27 | .208 | [-0.28, 0.06] |
| T × anxiety | -0.08 | 0.10 | -0.79 | .435 | [-0.27, 0.12] |
| E | 0.17 | 0.11 | 1.54 | .128 | [-0.05. 0.39] |
| Anxiety | -0.09 | 0.08 | -1.03 | .306 | [-0.25, 0.08] |
| E × anxiety | 0.04 | 0.11 | 0.37 | .709 | [-0.18, 0.26] |
| Perseverance |  |  |  |  |  |
| T | 0.07 | 0.04 | 1.51 | .134 | [-0.02, 0.15] |
| Anxiety | -0.16 | 0.04 | -3.75 | .000 | [-0.24, -0.08] |
| T × anxiety | 0.15 | 0.05 | 3.01 | .004 | [0.05, 0.25] |
| E | 0.02 | 0.06 | 0.32 | .752 | [-0.09, 0.13] |
| Anxiety | -0.15 | 0.04 | -3.61 | .001 | [-0.23, -0.07] |
| E × anxiety | -0.18 | 0.06 | -3.16 | .002 | [-0.29, -0.07] |

*Note.* B indicates unstandardized regression coefficients. CI =confidence interval. T = testosterone. E = estradiol. Endocrine group was entered as two dummy-coded covariates.
